# Mechanistic insights into the learning curve of cryoballoon ablation: ablation temperature mediates the effect of operator experience on radiation exposure

**DOI:** 10.3389/fcvm.2026.1931483

**Published:** 2026-08-27

**Authors:** Tian-Yu Li, Zhi-Ming Yu

**Affiliations:** Department of Cardiology, The Affiliated Wuxi People’s Hospital of Nanjing Medical University, Wuxi People’s Hospital, Wuxi Medical Center, Nanjing Medical University, Wuxi, China

**Keywords:** ablation temperature, atrial fibrillation, cryoballoon ablation, CUSUM analysis, learning curve, mediation analysis, pulmonary vein isolation, radiation exposure

## Abstract

**Background:**

Cryoballoon ablation (CBA) for atrial fibrillation (AF) is associated with reduced procedural radiation exposure as operator experience accumulates, yet the mechanistic underpinning of this improvement remains incompletely characterized. We hypothesized that ablation temperature parameters, reflecting balloon-tissue contact quality, serve as a quantifiable mediator linking operator experience to radiation dose.

**Methods:**

We retrospectively analyzed 804 consecutive patients undergoing first-time CBA for symptomatic AF at a single tertiary center. Cumulative sum (CUSUM) analysis identified learning curve inflection points. Patients were stratified into early (*n* = 385) and plateau (*n* = 419) phases. Multivariable linear regression, restricted cubic spline (RCS) analysis, and formal causal mediation analysis (bootstrap 1,000 iterations) were performed to evaluate worst pulmonary vein (PV) minimum temperature as a mediator between learning curve phase and log-transformed radiation dose.

**Results:**

CUSUM analysis identified learning curve inflection points at case 385 (procedural time), 301 (radiation dose), and 329 (fluoroscopy time). Compared with the early phase, median radiation dose in the plateau phase decreased by 15.4% (506 vs. 428 mGy, *p* < 0.001), procedural time by 27.0% (89 vs. 65 min, *p* < 0.001), and fluoroscopy time by 26.3% (19 vs. 14 min, *p* < 0.001). In multivariable analysis (adjusted R^2^ = 0.509), worst PV minimum temperature was an independent predictor of radiation dose (exp[β] = 1.012 per °C, 95% CI 1.008–1.017, *p* < 0.001), as was plateau phase (exp[β] = 0.895, 95% CI 0.847–0.946, *p* < 0.001). BMI exhibited a significant nonlinear association with radiation dose (*p* for nonlinearity < 0.001), with an inflection threshold near 25 kg/m^2^. Mediation analysis demonstrated that worst PV minimum temperature mediated 14.5% (95% CI 5.5%–35.4%) of the total learning curve effect on radiation dose (average causal mediation effect [ACME] *p* < 0.001; performed on 734 patients with complete mediator data).

**Conclusions:**

Greater operator experience was associated with lower radiation exposure and more favorable cryoablation temperature profiles. Worst PV nadir temperature statistically mediated a modest proportion of the association between learning curve phase and radiation dose, although most of the association remained unexplained by this temperature metric. These findings support a potential role of balloon–tissue contact quality in experience-related procedural improvement but should be interpreted as hypothesis-generating rather than evidence of a causal mechanism or improved clinical outcomes.

## Introduction

Atrial fibrillation (AF) affects more than 59 million individuals worldwide and is associated with substantial stroke risk, heart failure progression, and diminished quality of life (1, 2). Catheter ablation targeting pulmonary vein isolation (PVI) is the most effective rhythm-control strategy for symptomatic AF, and current guidelines recommend it as either a first- or second-line option depending on AF type and patient profile (3, 49).

Cryoballoon ablation (CBA) has emerged as the dominant single-shot PVI platform, validated against antiarrhythmic drug therapy in the landmark EARLY-AF, STOP-AF First, and Cryo-FIRST trials (4–6, 43, 50), and demonstrated to be non-inferior to point-by-point radiofrequency ablation in the FIRE AND ICE trial (7, 44). A principal attraction of CBA is its standardized single-shot delivery, which is perceived as reducing operator dependence compared with point-by-point techniques (8). Yet real-world registries and single-center series consistently demonstrate that procedural time, fluoroscopy duration, and radiation dose all decrease substantially as operator experience accumulates (9–12).

Radiation exposure during cardiac catheterization procedures is a recognized occupational and patient health risk. Cumulative operator exposure is associated with accelerated lens opacification, elevated skin cancer risk, and orthopedic disability, while patients incur stochastic cancer risk proportional to absorbed dose (13). Compared with radiofrequency ablation guided by electroanatomic mapping, CBA relies more heavily on fluoroscopy for balloon positioning and occlusion verification, making radiation reduction an area of particular clinical relevance (14, 15).

Despite consistent documentation of learning curve radiation benefits, the mechanism through which operator experience translates to reduced radiation exposure in CBA has not been formally quantified. Balloon-tissue contact quality during CBA is reflected by the minimum temperature achieved during each cryofreeze. Colder nadir temperatures indicate more complete PV occlusion and efficient cryothermal delivery, and have been associated with more durable PVI, fewer PV reconnections, and lower AF recurrence rates (16–19, 39). A plausible mechanistic chain therefore exists: as operators gain experience, they achieve more precise balloon positioning and consistent PV occlusion, yielding lower freeze temperatures, fewer repositioning attempts, and ultimately reduced fluoroscopy and radiation exposure. This pathway, however, has never been formally tested using causal mediation analysis.

In the present study, we analyzed 804 consecutive CBA procedures at a single high-volume center to: (1) quantify the learning curve using CUSUM analysis across three procedural efficiency metrics; (2) identify independent predictors of radiation dose with particular attention to ablation temperature parameters; (3) apply causal mediation analysis to formally quantify the proportion of the learning curve radiation benefit explained by worst PV minimum temperature; and (4) characterize the nonlinear relationship between BMI and radiation exposure. To our knowledge, this is the first study to employ formal causal mediation analysis to decompose the learning curve effect on radiation dose in CBA.

## Methods

### Study population and design

We retrospectively analyzed 804 consecutive patients (462 men [57.5%] and 342 women [42.5%]) who underwent first-time second-generation or fourth-generation CBA (Arctic Front Advance, Medtronic, Dublin, Ireland) for symptomatic drug-refractory paroxysmal or persistent AF at a single tertiary electrophysiology center between January 2019 and December 2025.Procedures were performed by a single primary operator throughout the study period. Patients with prior ablation procedures, structural heart disease requiring concomitant intervention, or incomplete procedural temperature records were excluded. The study was approved by the Ethics Committee of Wuxi People's Hospital (approval number: KY26162), and the requirement for individual informed consent was waived given the retrospective nature of the analysis.

### Cryoballoon ablation procedure

All procedures were performed under conscious sedation. A single transseptal puncture was guided by fluoroscopy, and systemic anticoagulation was maintained throughout with activated clotting time targets of 300 s–350 s. The 28 mm cryoballoon was advanced via a steerable 15-Fr deflectable sheath (FlexCath Advance, Medtronic). Each PV was assessed for occlusion by contrast injection through the distal lumen under fluoroscopy, as described in consensus best practice recommendations (20, 21). A single freeze application of 180 s–240 s was delivered per PV, with an additional application in cases of incomplete isolation or nadir temperature above −35 °C. Continuous phrenic nerve monitoring was performed during right-sided PV applications. During right-sided PV cryoablation, the right phrenic nerve was continuously paced from the superior vena cava using a diagnostic catheter, while diaphragmatic contraction was monitored by manual abdominal palpation throughout the freeze application. Cryoenergy delivery was immediately terminated if weakening or loss of diaphragmatic contraction was observed (40–42). The minimum (nadir) temperature achieved during the first freeze application at each of the four PVs was recorded from the cryoconsole log.

### Variable definitions and data collection

Primary outcome: log-transformed radiation dose (mGy). Total procedural time (minutes, skin-to-skin) and fluoroscopy time (minutes) were secondary outcomes analyzed in supplementary multivariable models. The key candidate mediator was *worst PV minimum temperature*, defined as the highest (least negative) nadir temperature among the four PVs in each procedure, representing the most challenging PV in that case. BMI values exceeding 60 kg/m^2^ (*n* = 11, judged as data entry errors) and temperature values outside the range of −10 °C to −80 °C (*n* = 7) were set to missing prior to analysis. All remaining variables were extracted from the electronic catheterization laboratory system and clinical records.

### CUSUM analysis and learning curve phase definition

Learning curve inflection points were identified by cumulative sum (CUSUM) analysis, computed as S(n) = Σᵢ₌₁ⁿ (Xᵢ − $\bar{X}$), where Xᵢ is the procedural metric for the i-th case and $\bar{X}$ is the cohort mean. The inflection point was defined as the case number at which S(n) reached its maximum. This methodology has been validated in electrophysiology learning curve studies (22, 23). The inflection point for procedural time (case 385) was selected as the primary case-count definition for the early vs. plateau phase dichotomy, as it showed the strongest concordance with the other two metrics.

### Statistical analysis

Variable selection for multivariable modeling was performed using LASSO regression with 10-fold cross-validation across 20 multiply-imputed datasets (*mice* package, R; lambda.1se criterion), yielding 11 candidate predictors: age, BMI, AF type, stroke history, left atrial diameter (LAD), ejection fraction (EF), heparin dose, worst PV minimum temperature, phase, sex, and hyperlipidemia. Multivariable linear regression with log-transformed radiation dose as the dependent variable was then performed. Variance inflation factors (VIF) were all below 2.0, indicating no meaningful multicollinearity. Coefficients are reported as exp(β) ratios for clinical interpretability. Serum creatinine differed between groups in univariable comparison but was not retained by LASSO selection across the 20 multiply-imputed datasets, likely reflecting collinearity with other metabolic predictors and limited independent contribution to radiation dose variance after adjustment for BMI and procedural complexity surrogates; it was therefore excluded from the final model.

Restricted cubic spline (RCS) analysis was performed for five continuous predictors (age, BMI, LAD, AF duration, mean PV minimum temperature) to evaluate nonlinear associations with log-transformed radiation dose. Causal mediation analysis was performed using the R *mediation* package (24) with worst PV minimum temperature as mediator, learning curve phase as exposure, and log-transformed radiation dose as outcome, adjusting for all covariates. Bootstrap resampling (1,000 iterations) estimated 95% confidence intervals for the average causal mediation effect (ACME), average direct effect (ADE), total effect, and proportion mediated.

Sensitivity analyses were conducted in four scenarios: (1) excluding 2019 cases (first calendar year); (2) excluding patients with radiation dose above the 99th percentile; (3) paroxysmal AF only; and (4) persistent AF only. Subgroup analyses examined the effect of worst PV minimum temperature on radiation dose across 12 predefined strata (sex, AF type, age, BMI, LAD, and learning curve phase). Supplementary multivariable regression models with log-transformed procedural time and fluoroscopy time as outcomes were performed to assess consistency across endpoints. All analyses were conducted in R version 4.5.2 (R Foundation for Statistical Computing, Vienna, Austria). A two-sided *p* < 0.05 was considered statistically significant.

## Results

### Baseline characteristics

A total of 804 patients (mean age 62.2 ± 10.3 years, 57.5% male) were included. Paroxysmal AF was present in 474 (59.0%) and persistent AF in 330 (41.0%). Baseline characteristics stratified by learning curve phase are presented in Table 1. The two phases were largely comparable. AF type (*p* = 0.940), LAD (*p* = 0.701), EF (*p* = 0.456), sex (*p* = 0.087), and most comorbidities showed no significant between-group differences. Notably, the plateau phase had a modestly higher prevalence of diabetes mellitus (18.9% vs. 11.9%, *p* = 0.009) and slightly older mean age (63.1 ± 9.8 vs. 61.3 ± 10.7 years, *p* = 0.013), indicating that patients in the more experienced phase were not systematically simpler.

**Table 1:** Baseline clinical and procedural characteristics stratified by learning curve phase.

Variable	Overall (*n* = 804)	Early phase (*n* = 385)	Plateau phase (*n* = 419)	*P* value
Age, years, mean (SD)	62.2 (10.3)	61.3 (10.7)	63.1 (9.8)	0.013
Male sex, *n* (%)	462 (57.5)	233 (60.5)	229 (54.7)	0.087
BMI, kg/m^2^, mean (SD)	25.2 (3.3)	25.0 (3.5)	25.3 (3.2)	0.146
Persistent AF, *n* (%)	330 (41.0)	157 (40.8)	173 (41.3)	0.940
AF duration, months, median [IQR]	24 [8, 60]	24 [7, 60]	24 [9, 72]	0.426
Hypertension, *n* (%)	486 (60.4)	232 (60.3)	254 (60.6)	0.974
Diabetes mellitus, *n* (%)	125 (15.5)	46 (11.9)	79 (18.9)	0.009
Coronary artery disease, *n* (%)	94 (11.7)	46 (11.9)	48 (11.5)	0.915
Stroke history, *n* (%)	70 (8.7)	33 (8.6)	37 (8.8)	0.996
Hyperlipidemia, *n* (%)	103 (12.8)	42 (10.9)	61 (14.6)	0.150
Left atrial diameter, mm, mean (SD)	40.8 (5.6)	40.8 (5.8)	40.7 (5.3)	0.701
Ejection fraction, %, mean (SD)	62.4 (3.9)	62.5 (4.1)	62.3 (3.8)	0.456
Heparin dose, U, median [IQR]	8,000 [7,000, 10,000]	8,000 [7,000, 10,000]	8,000 [6,000, 9,000]	<0.001
Creatinine, µmol/L, mean (SD)	75.1 (17.1)	78.0 (16.5)	72.5 (17.2)	<0.001
Procedure time, min, median [IQR]	75 [65, 90]	89 [80, 100]	65 [60, 75]	<0.001
Radiation dose, mGy, median [IQR]	465 [337, 644]	506 [363, 709]	428 [318, 610]	<0.001
Fluoroscopy time, min, median [IQR]	16 [13, 20]	19 [16, 23]	14 [12, 17]	<0.001
Mean PV min temperature, °C, mean (SD)	−45.8 (4.7)	−45.3 (4.7)	−46.3 (4.6)	0.002
Worst PV min temperature, °C, mean (SD)	−38.6 (6.3)	−37.8 (6.5)	−39.4 (6.0)	<0.001

Values are mean ± SD, median [IQR], or *n* (%). *P* values: Student’s *t*-test, Mann–Whitney *U-*test, or chi-square test as appropriate.

BMI, body mass index; AF, atrial fibrillation; PV, pulmonary vein.

### Learning curve analysis

**Figure 1:**
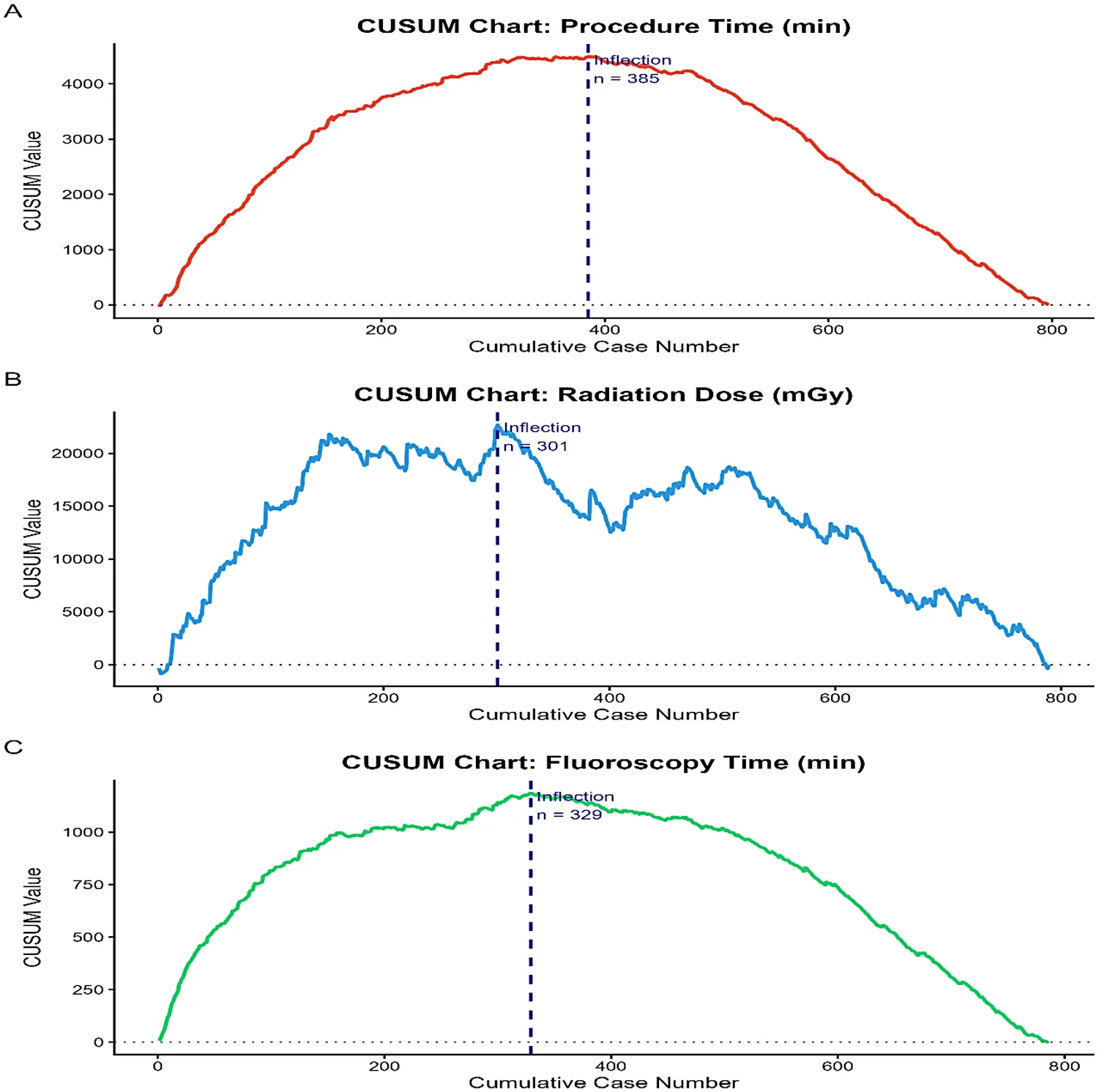
Cumulative sum (CUSUM) analysis of three procedural efficiency metrics across 804 consecutive cryoballoon ablation cases. **(A)** Procedural time, inflection at case 385. **(B)** Radiation dose, inflection at case 301. **(C)** Fluoroscopy time, inflection at case 329. The concordance of inflection points across all three metrics indicates a coherent, global improvement in procedural performance. Vertical dashed line marks the CUSUM peak in each panel.

CUSUM analysis revealed consistent learning curve inflection points across all three procedural efficiency metrics: case 385 for procedural time, case 301 for radiation dose, and case 329 for fluoroscopy time (Figure 1). The temporal concordance of these three CUSUM curves indicates a global, coherent improvement in procedural performance rather than improvement restricted to a single metric. This pattern of concordance has been noted in learning curve analyses of other novel ablation platforms (23, 25).

**Figure 2:**
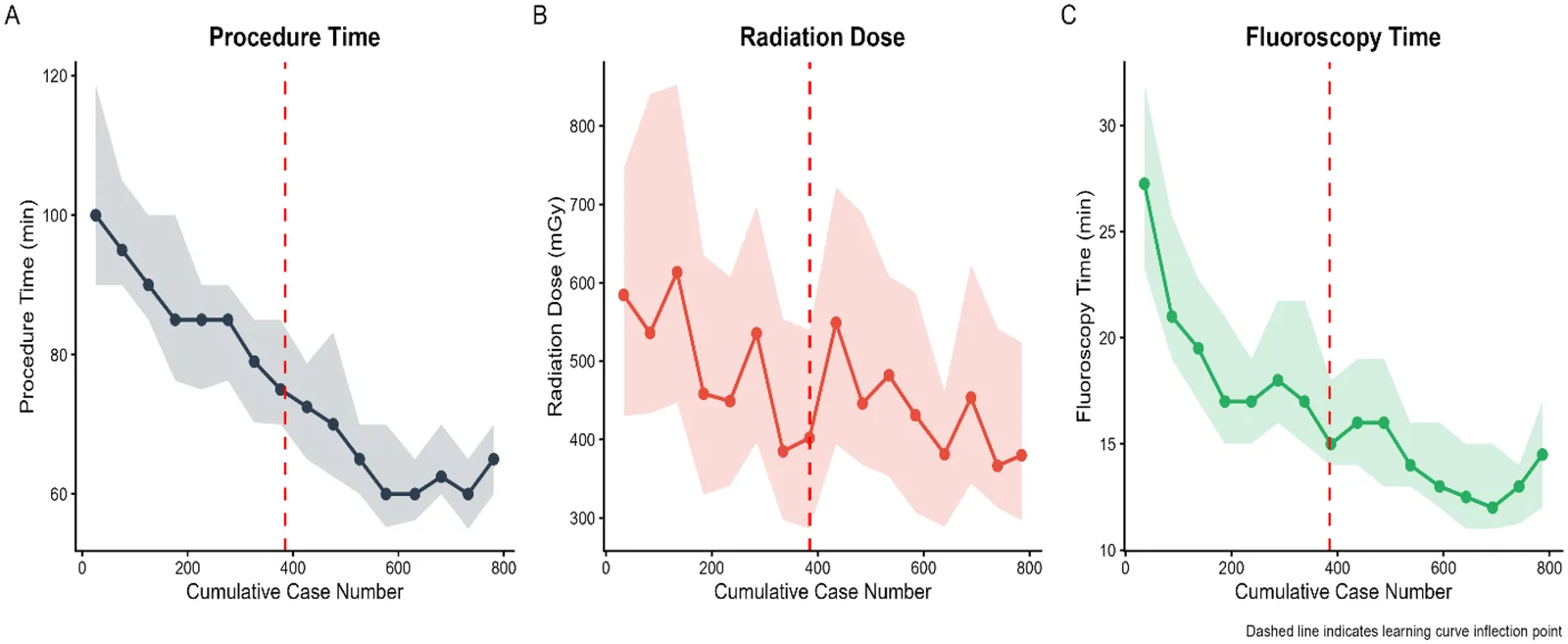
Segmented trend analysis of procedural outcomes across 804 consecutive cases. **(A)** Procedural time (minutes). **(B)** Radiation dose (mGy). **(C)** Fluoroscopy time (minutes). Each data point represents one procedure; the smooth line represents a LOESS-fitted trend. Vertical dashed line marks the primary learning curve inflection point (case 385).

Segmented trend analysis confirmed a continuous, progressive decline in all three outcomes over the 804 cases (Figure 2). Procedural time showed a smooth decrease from approximately 100 min in the earliest cases to approximately 60 min by the plateau phase. Radiation dose demonstrated a similar downward trajectory, though with substantial case-level variability (IQR in the early phase: 363–709 mGy), anticipating the importance of patient-level predictors beyond operator experience alone. Fluoroscopy time showed the most rapid early decrease, approaching a steady state within the first 100–150 cases.

When comparing early vs. plateau phase directly (Table 2), median radiation dose declined from 506 mGy (IQR 363–709) to 428 mGy (IQR 318–610), a 15.4% relative reduction (*p* < 0.001). Median procedural time decreased by 27.0% (89–65 min, *p* < 0.001), and median fluoroscopy time by 26.3% (19–14 min, *p* < 0.001). Critically, worst PV minimum temperature was significantly lower (more negative) in the plateau phase (−39.4 ± 6.0 °C vs. −37.8 ± 6.5 °C, *p* < 0.001), as was mean PV minimum temperature (−46.3 ± 4.6 °C vs. −45.3 ± 4.7 °C, *p* = 0.002). These findings suggest that accumulated experience translated to improved cryothermal delivery quality, consistent with observations that experienced operators achieve colder nadir temperatures in anatomically challenging veins (26).

**Table 2:** Procedural outcomes and ablation temperature parameters by learning curve phase.

Outcome	Early phase (*n* = 385)	Plateau phase (*n* = 419)	*P* value
Procedure time, min, median [IQR]	89 [80–100]	65 [60–75]	<0.001
Radiation dose, mGy, median [IQR]	506 [363–709]	428 [318–610]	<0.001
Fluoroscopy time, min, median [IQR]	19 [16–23]	14 [12–17]	<0.001
Heparin dose, U, median [IQR]	8,000 [7,000–10,000]	8,000 [6,000–9,000]	<0.001
Worst PV min temperature, °C, mean (SD)	−37.8 ± 6.5	−39.4 ± 6.0	<0.001
Mean PV min temperature, °C, mean (SD)	−45.3 ± 4.7	−46.3 ± 4.6	0.002

Values are median [IQR] or mean ± SD. PV, pulmonary vein.

### Multivariable predictors of radiation dose

**Figure 3:**
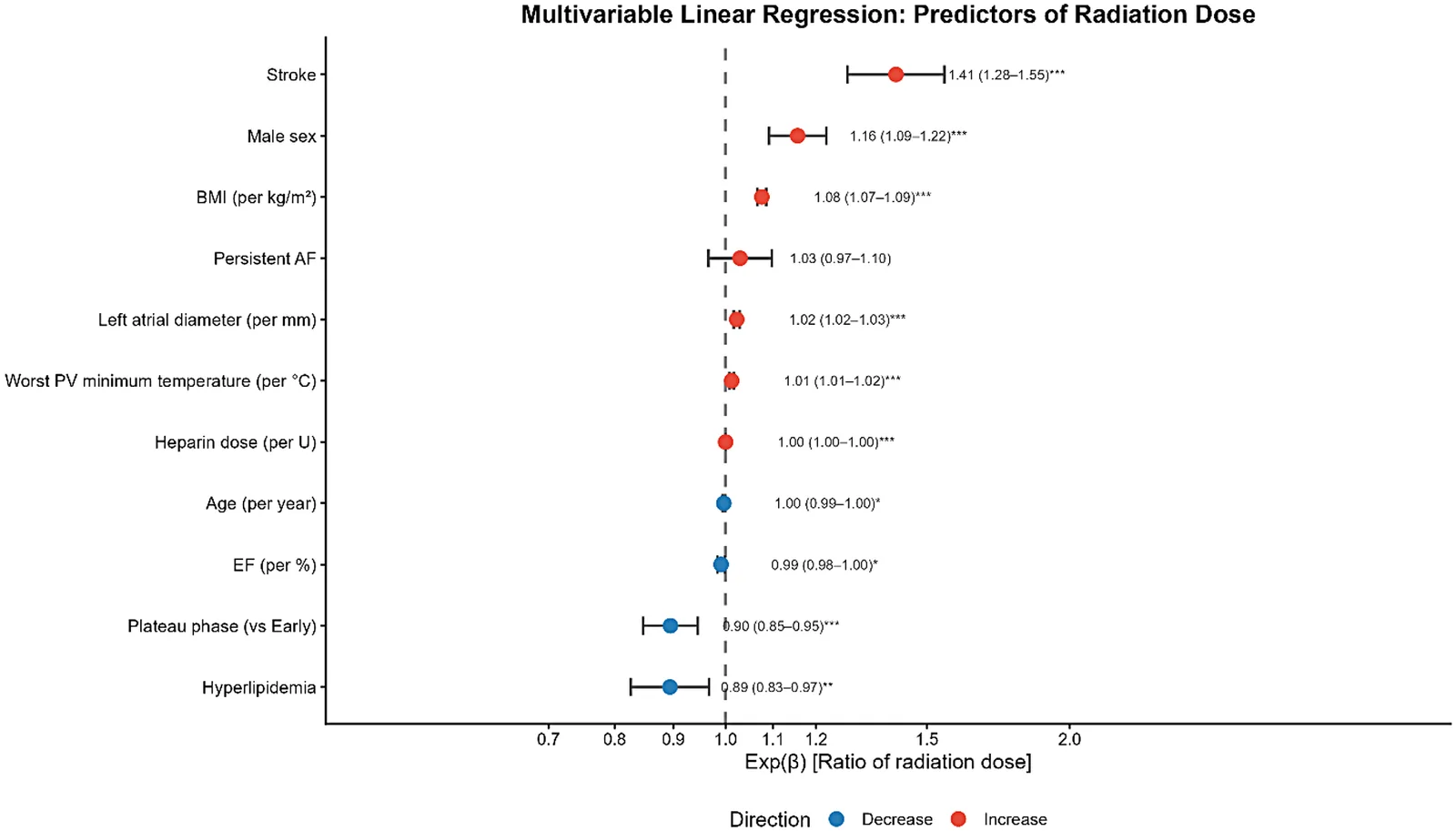
Forest plot of multivariable linear regression predictors of log-transformed radiation dose (adjusted R^2^ = 0.509). Coefficients are expressed as exp(β) ratios. Vertical dashed reference line at exp(β) = 1.0. Significance: *p < 0.05; **p < 0.01; ***p < 0.001. BMI, body mass index; EF, ejection fraction; PV, pulmonary vein; LAD, left atrial diameter.

The multivariable linear regression model accounted for 50.9% of variance in log-transformed radiation dose (adjusted R^2^ = 0.509, all VIF<2.0; Figure 3). The strongest predictor was stroke history (exp[β] = 1.409, 95% CI 1.278–1.554, *p* < 0.001). Male sex (exp[β] = 1.156, *p* < 0.001), BMI (exp[*β*] = 1.076 per kg/m^2^, *p* < 0.001), LAD (exp[*β*] = 1.023 per mm, *p* < 0.001), and heparin dose (*p* < 0.001) were also independent positive predictors. These associations are broadly consistent with prior work documenting the radiation-increasing effects of obesity, larger left atrial dimensions, and procedural complexity surrogates in CBA (14, 27).

Two variables of central mechanistic interest were independently significant. First, worst PV minimum temperature was a significant positive predictor: each degree Celsius increase (i.e., less negative, indicating poorer balloon contact quality) was associated with a 1.2% higher radiation dose (exp[β] = 1.012 per °C, 95% CI 1.008–1.017, *p* < 0.001) after adjusting for all other predictors. Second, plateau phase was independently associated with lower radiation dose (exp[β] = 0.895, 95% CI 0.847–0.946, *p* < 0.001), representing a 10.5% direct reduction in radiation beyond what was accounted for by temperature alone. An important negative finding was that persistent AF type was not an independent predictor of radiation dose after covariate adjustment (*p* = 0.369), despite its significant association in univariable analysis, indicating that LAD rather than AF type itself is the operative driving factor. Hyperlipidemia showed an inverse association (exp[β] = 0.894, *p* = 0.005), likely reflecting selection or confounding rather than a direct biological mechanism.

### Nonlinear association between BMI and radiation dose

**Figure 4:**
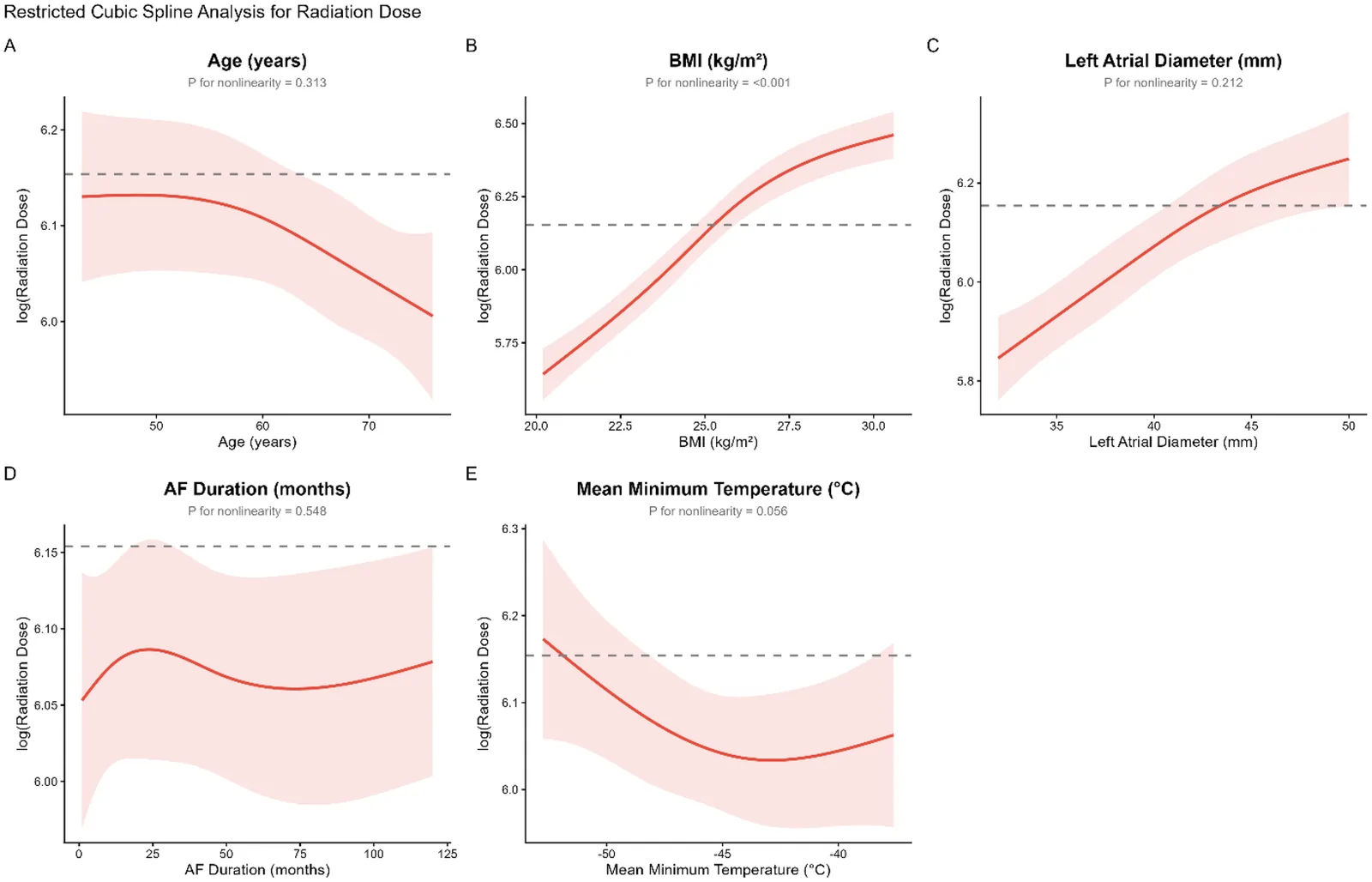
Restricted cubic spline (RCS) analysis of continuous predictors and log-transformed radiation dose. **(A)** Age. **(B)** BMI. **(C)** Left atrial diameter. **(D)** AF duration. **(E)** Mean PV minimum temperature. *P* values for nonlinearity are displayed in each panel. Only BMI showed a statistically significant nonlinear relationship (*p* < 0.001).

RCS analysis identified a statistically significant nonlinear relationship between BMI and log-transformed radiation dose (*p* for nonlinearity<0.001; Figure 4), with a threshold effect evident near a BMI of 25 kg/m^2^. Below this threshold, the increase in radiation with rising BMI was relatively gradual. Above it, the relationship steepened markedly, suggesting that x-ray scatter and dose-rate auto-compensation algorithms create a superlinear radiation burden in patients with higher BMI values. This nonlinear pattern has not been systematically characterized in prior CBA radiation studies, which have generally modeled BMI as a linear predictor (14, 27, 28). Age, LAD, AF duration, and mean PV minimum temperature all showed linear associations (all *p* for nonlinearity > 0.05).

### Mediation analysis

**Figure 5:**
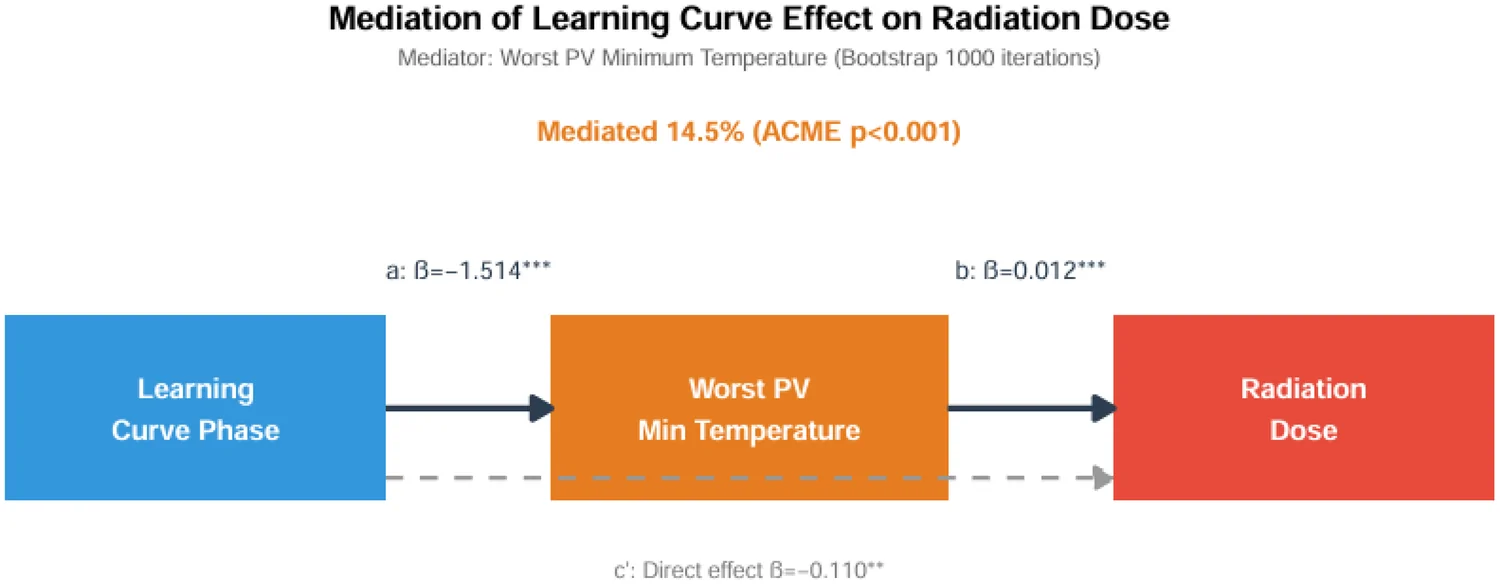
Causal mediation pathway diagram. Path a: effect of learning curve phase on worst PV minimum temperature (β = −1.514, *p* < 0.001). Path b: independent effect of worst PV minimum temperature on log-transformed radiation dose (β = 0.012, *p* < 0.001). Path c’: direct effect of learning curve phase on radiation dose after adjusting for the mediator (β = −0.110, *p* = 0.004). The proportion of the total effect mediated by temperature is 14.5% (ACME *p* < 0.001; bootstrap, 1,000 iterations).

**Figure 6:**
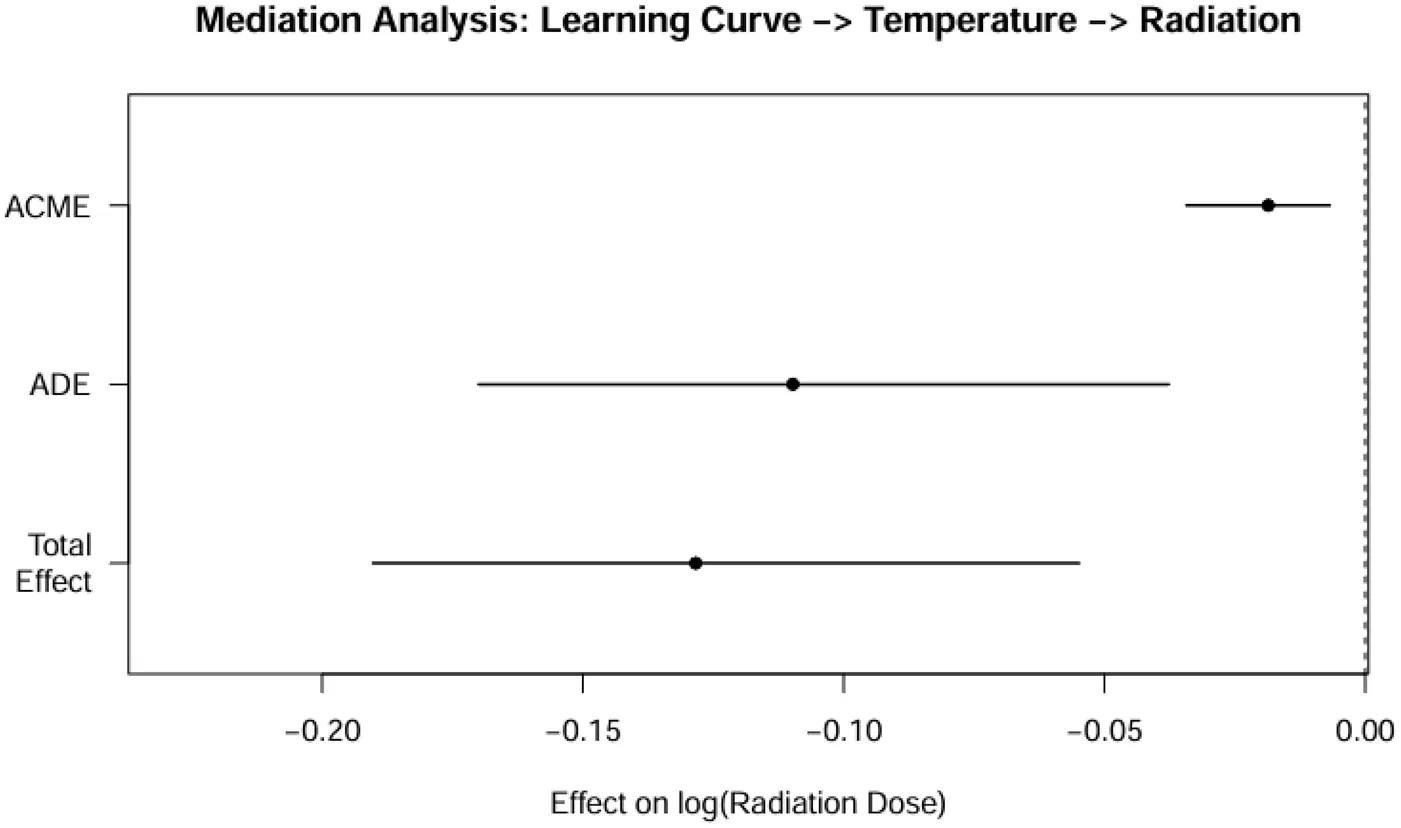
Effect estimates from causal mediation analysis on log-transformed radiation dose. Bars represent the total effect **(c)**, average direct effect (ADE), and average causal mediation effect (ACME). Error bars indicate 95% bootstrap confidence intervals.

Formal causal mediation analysis confirmed that worst PV minimum temperature partially mediated the relationship between learning curve phase and log-transformed radiation dose (Table 3, Figures 5,6). The total effect of plateau vs. early phase was −0.128 (95% CI −0.190 to −0.055, *p* < 0.001). The ACME transmitted through worst PV minimum temperature was −0.019 (95% CI −0.034 to −0.007, *p* < 0.001), accounting for 14.5% of the total effect (95% CI 5.5%–35.4%). The average direct effect was −0.110 (95% CI −0.170 to −0.038, *p* = 0.004), indicating that the majority of the learning curve benefit (85.5%) reflects mechanisms beyond temperature improvement, including workflow optimization, fluoroscopy technique refinement, and team-level proficiency gains. Mediation analysis was conducted on a complete-case sample (*n* = 734) due to the requirements of the mediation package. Sensitivity analysis for unmeasured confounding indicated directional stability of the ACME at rho values up to 0.15.

**Table 3:** Causal mediation analysis results.

Effect	Estimate	*P* value	95% CI lower	95% CI upper
Total effect (c)	−0.128	<0.001	−0.190	−0.055
Direct effect (c’)	−0.110	0.004	−0.170	−0.038
Indirect effect via worst PV min temp (ACME)	−0.019	<0.001	−0.034	−0.007
Proportion mediated	14.5%	—	5.5%	35.4%

Worst PV minimum temperature as mediator of the effect of learning curve phase (early vs. plateau) on log-transformed radiation dose, adjusting for age, sex, BMI, AF type, stroke, LAD, EF, heparin dose, and hyperlipidemia. Bootstrap 95% confidence intervals (1,000 resamples); *n* = 734 complete cases.

ACME, average causal mediation effect.

### Consistency across procedural outcomes

Supplementary multivariable regression models with log-transformed procedural time and fluoroscopy time as outcomes confirmed the consistency of findings (Supplementary Tables S1,S2). In both models, worst PV minimum temperature and plateau phase were significant independent predictors (all *p* < 0.001). Plateau phase was associated with a 25.0% reduction in log-procedural time *(*β = −0.250, *p* < 0.001) and a 23.5% reduction in log-fluoroscopy time (β = −0.268, *p* < 0.001). Worst PV minimum temperature independently predicted longer procedural time (*p* = 3.7 × 10⁻^8^) and longer fluoroscopy time (*p* = 4.6 × 10⁻^11^), providing cross-outcome corroboration of the mechanistic pathway.

### Sensitivity and subgroup analyses

Sensitivity analyses across four scenarios confirmed robustness of the primary findings (Table 4). Worst PV minimum temperature remained a significant independent predictor in all scenarios except the persistent AF-only subgroup (exp[β] = 1.006, *p* = 0.140). This attenuation is discussed further below. Phase was consistently significant across all four sensitivity analyses (all *p* < 0.05). Subgroup analyses across 12 patient strata showed broadly consistent point estimates, with no statistically significant interactions detected (Supplementary Figure S3). These findings suggest no clear evidence of effect modification within the present cohort, although the analyses were exploratory and may have had limited power to detect subgroup differences (29).

**Table 4:** Sensitivity analyses for worst PV minimum temperature and learning curve phase as predictors of log-transformed radiation dose.

Analysis	n	Worst PV temp exp(β) (95% CI)	*p* (worst temp)	Phase exp(β) (95% CI)	*p* (phase)
Primary analysis	804	1.012 (1.008–1.017)	<0.001	0.895 (0.847–0.946)	<0.001
Excluding 2019 cases	734	1.012 (1.008–1.017)	<0.001	0.895 (0.847–0.946)	<0.001
Excluding extreme radiation (>99th pct)	719	1.011 (1.007–1.016)	<0.001	0.913 (0.866–0.963)	<0.001
Paroxysmal AF only	474	1.016 (1.010–1.022)	<0.001	0.889 (0.829–0.953)	<0.001
Persistent AF only	330	1.006 (0.998–1.013)	0.140	0.903 (0.824–0.989)	0.028

All models include identical covariates as the primary multivariable model.

## Discussion

The principal finding of this study is that greater operator experience was associated with both lower radiation exposure and more favorable cryoablation temperature profiles, and that worst PV nadir temperature statistically accounted for a modest proportion of this association. To our knowledge, this is the first study to apply formal mediation analysis to the CBA learning curve. Given the retrospective observational design, however, the mediation findings should be interpreted as evidence of a statistically compatible pathway rather than proof of a causal biological or procedural mechanism.

### Mechanistic Interpretation of the Learning Curve

Existing single-center and registry data have consistently documented that procedural time, fluoroscopy time, and radiation dose decrease with operator experience in CBA (9–12, 23). However, the mechanistic basis of these improvements has remained largely speculative. Our mediation analysis provides the first formal quantification of one such pathway: worst PV minimum temperature accounts for approximately 14.5% of the total learning curve effect on radiation dose (ACME *p* < 0.001; directionally stable across all sensitivity scenarios). The magnitude of this proportion warrants contextual interpretation. In causal mediation studies of complex procedural interventions, proportion-mediated estimates in the range of 10%–20% are common and clinically informative when the mediator is objectively measured, statistically robust, and mechanistically plausible—all of which apply here (30). Critically, CBA is among the most operator-constrained ablation platforms available: the single-shot delivery format, fixed balloon geometry, and standardized freeze protocol leave fewer modifiable technical variables than point-by-point radiofrequency ablation (45, 48). Within this tightly constrained framework, the identification of any single measurable mediator that independently and reproducibly explains a statistically significant fraction of the learning curve benefit represents a meaningful advance (46, 47). Our mediation analysis estimated that worst PV minimum temperature accounted for approximately 14.5% of the association between learning curve phase and radiation dose. Although statistically significant, this proportion should be interpreted cautiously. Cryoballoon procedural efficiency is inherently multifactorial, and nadir temperature represents only one measurable feature related to balloon–tissue contact and PV occlusion. In an observational setting, mediation analysis cannot establish that changes in nadir temperature causally produced the observed reduction in radiation exposure. Rather, the findings indicate that variation in worst PV minimum temperature is statistically consistent with one component of the broader experience-related improvement in procedural performance. Importantly, approximately 85% of the observed association was not accounted for by this temperature metric, underscoring the likely contribution of multiple additional factors. These may include refinement of fluoroscopy technique, greater familiarity with PV anatomy, improved transseptal and balloon manipulation, more efficient procedural sequencing, operator decision-making, and increasing coordination of the electrophysiology laboratory team. These factors were not comprehensively measured in the present study and therefore cannot be ranked according to their relative contribution. Future prospective multi-operator studies incorporating detailed procedural metrics, such as fluoroscopy settings, time-to-isolation, balloon repositioning attempts, contrast use, and transseptal access time, will be needed to more completely characterize the determinants of experience-related reductions in radiation exposure. Fluoroscopy technique refinement—including adoption of lower frame rates, collimation discipline, and reduced cine acquisition—likely contributes the largest unmeasured fraction, as these behaviors are acquired incrementally through experience but leave no temperature signature (31–33). Workflow sequencing improvements, including faster transseptal access and more efficient balloon exchanges, reduce total procedural time and thereby fluoroscopy duration independent of occlusion quality (20, 21). Team-level coordination gains, including anticipatory sheath management and contrast preparation, further compress procedural length. Incorporating fluoroscopy frame-rate logs, time-to-isolation, and transseptal access duration as candidate mediators in future multi-operator cohorts would allow a more complete mechanistic decomposition of the learning curve radiation benefit.

### Stroke history as the strongest radiation predictor

The strongest independent predictor of radiation dose in the multivariable model was prior stroke history (exp[β] = 1.409). This finding likely reflects two non-mutually exclusive mechanisms. First, patients with prior stroke require more stringent intraoperative anticoagulation surveillance and procedural caution, which may prolong balloon positioning attempts and fluoroscopy use due to interruptions for activated clotting time checks and heightened awareness of thromboembolic risk. Second, and perhaps more importantly, stroke history may serve as a surrogate marker for advanced left atrial structural remodeling. Data from the DECAAF II trial have demonstrated that a history of stroke is independently associated with a higher burden of left atrial fibrosis on late gadolinium enhancement MRI in persistent AF patients (34), suggesting that stroke history identifies a subset of patients with more advanced atrial myopathy and greater anatomical complexity. Greater left atrial fibrosis is associated with pulmonary vein anatomical distortion and reduced ostial compliance, both of which impair reliable balloon occlusion and necessitate more frequent repositioning maneuvers, each requiring fluoroscopic verification (35). Together, these two mechanisms—procedural caution and anatomical complexity—plausibly account for the disproportionate radiation burden observed in this subgroup and highlight stroke history as a clinically actionable preprocedural risk marker for elevated radiation exposure in CBA.

### Worst PV minimum temperature as a “weakest link” metric

A key observation is that *worst* PV minimum temperature, not *mean* PV minimum temperature, emerged as the significant predictor in multivariable analysis (mean temperature: *p* = 0.125 univariable, not entering the final model). This aligns with a barrel-principle logic: radiation burden in a CBA procedure is disproportionately driven by the most technically demanding PV. The right inferior pulmonary vein (RIPV) is anatomically the most challenging vein to occlude due to its posterior-inferior orientation relative to the transseptal puncture site (36, 37). Prior learning curve data showed that hands-on assistance from senior operators was required most frequently for the RIPV (9), consistent with our finding that this vein most commonly determines worst PV temperature. A single challenging PV requiring multiple repositionings, repeated contrast injections, and prolonged fluoroscopic monitoring dominates the session-level radiation budget. From an educational perspective, worst PV temperature may represent a readily available procedural metric that could potentially complement other indicators of procedural performance during operator training. Prospective studies are required to determine whether targeted use of this metric improves procedural efficiency, radiation exposure, or clinical outcomes.

### BMI and the nonlinear radiation threshold

The RCS analysis identified a nonlinear BMI-radiation relationship with a threshold near 25 kg/m^2^. Although the linear association between obesity and elevated radiation exposure in CBA is well documented (14, 27, 28), the existence of a threshold effect has not been systematically characterized. Above 25 kg/m^2^, adipose tissue attenuates x-rays and triggers automatic dose-rate compensation algorithms that increase fluoroscopic output nonlinearly, rather than proportionally. Obesity was the most prevalent modifiable predictor in our cohort (BMI ≥ 25: *n* = 419, 52.1%), and this nonlinear pattern may have practical relevance for radiation-reduction strategies in patients with higher BMI. However, the observed threshold was derived from a single-center cohort and should be externally validated before being used to guide operator assignment or procedural protocols.

Association in Paroxysmal and Persistent AF Subgroups In stratified analyses, the association between worst PV minimum temperature and radiation dose remained statistically significant in patients with paroxysmal AF but did not reach statistical significance in those with persistent AF. However, the interaction between AF type and worst PV minimum temperature was not statistically significant. Therefore, these subgroup findings should not be interpreted as evidence of a differential effect according to AF type. The weaker estimate in the persistent AF subgroup may reflect reduced precision, greater anatomical or procedural heterogeneity, or other unmeasured factors and should be considered exploratory. First, persistent AF is associated with progressive left atrial enlargement and structural remodeling, which increases the variability of PV ostial anatomy and impairs consistent balloon-tissue contact, introducing additional anatomically driven radiation determinants that are independent of operator skill as indexed by nadir temperature (35). Second, persistent AF procedures may require additional non-PVI ablation of the left atrial posterior wall or roof, generating radiation exposure that is structurally unrelated to PV occlusion quality and thereby diluting the temperature signal. This increased procedural heterogeneity introduces multiple competing radiation drivers that collectively attenuate the explanatory power of any single temperature metric. Importantly, after covariate adjustment including LAD, persistent AF type itself was not an independent radiation predictor, replicating prior observations that the perceived radiation risk of persistent AF is largely mediated by atrial dimensions rather than AF subtype *per se*.

### The 385-case learning curve inflection point

Our CUSUM-defined inflection point (cases 301–385) is substantially higher than the 20–30 case threshold reported in earlier second-generation CBA learning curve analyses that focused primarily on PVI success rates and acute procedural safety (9). This apparent discrepancy is methodologically important. Acute efficacy metrics such as PVI success rates plateau early because even novice operators can achieve adequate balloon occlusion in most cases. Radiation optimization, by contrast, requires the accumulated maturation of fine motor skills, workflow sequencing, fluoroscopy frame-rate discipline, and whole-team coordination that continues to evolve over hundreds of procedures. Our findings resonate with a recent JCE study documenting ongoing performance improvement beyond 100 cases with novel cryoballoon platforms (25), and with registry data showing that operator volume correlates with complication rates across a broad range of ablation procedures (13). The implication is that learning curve assessment for radiation endpoints should not be conflated with assessment for efficacy endpoints; these represent distinct performance dimensions.

### Limitations

Several limitations warrant acknowledgment. This is a single-center retrospective study by a single operator. The single-operator design eliminates inter-operator variability as a confounding factor, which strengthens the internal validity of the CUSUM inflection estimate; however, the absolute case-number threshold of 385 cases reflects the experience trajectory of one operator at one institution and should not be interpreted as a universal benchmark. Learning curve inflection points in multi-operator settings or lower-volume centers are likely to differ substantially, and the magnitude of radiation reduction may not be directly generalizable to other institutions or operators. The mediation analysis rests on the assumption of sequential ignorability (no unmeasured confounders of the mediator-outcome pathway given observed covariates), which cannot be verified in observational data; sensitivity analyses provided some reassurance of directional robustness. The proportion mediated (14.5%) captures only the temperature pathway; the wide confidence interval (5.5%–35.4%) is consistent with the inherent variability of bootstrap-based mediation estimates in observational studies and reflects the exploratory nature of this analysis. Importantly, the ACME was statistically significant and directionally robust across all sensitivity scenarios, and other mediators remain unmeasured. Time-to-isolation data were unavailable for 76.4% of cases and could not be included as an additional candidate mediator. TTI can only be recorded when real-time pulmonary vein electrical signals are detectable during the freeze application. In cases where the Achieve mapping catheter fails to capture stable electrograms, or where PV isolation occurs without a discernible signal transition, TTI cannot be documented regardless of operator intent. This represents a physiological and technical limitation inherent to cryoballoon ablation rather than a systematic data collection failure (38). In addition, procedural efficiency and radiation exposure are determined by multiple operator-, patient-, anatomical-, equipment-, and workflow-related factors. The present analysis evaluated only a subset of these determinants, and the estimated mediation proportion should therefore not be interpreted as a comprehensive mechanistic decomposition of the learning curve. Moreover, our study focused on procedural efficiency and radiation exposure and did not evaluate longer-term clinical outcomes, including arrhythmia recurrence, repeat ablation, durable pulmonary vein isolation, or late procedure-related complications. Consequently, although lower radiation exposure is itself a desirable procedural objective, the present findings do not demonstrate that the observed learning-curve or temperature-related differences translate into improved rhythm-control efficacy or other clinical outcomes. These associations require validation in prospective, multicenter, multi-operator cohorts with systematic clinical follow-up.

## Conclusion

In this single-center, single-operator cohort, greater operator experience was associated with lower radiation exposure during CBA. Worst PV nadir temperature was independently associated with radiation dose and statistically accounted for a modest proportion of the association between learning curve phase and radiation exposure. These findings are consistent with a potential contribution of improved balloon–tissue contact to experience-related procedural improvement, while also indicating that most of the learning-curve association is likely attributable to other measured and unmeasured factors. Worst PV nadir temperature may represent a readily available procedural metric worthy of further evaluation, but prospective multicenter studies are needed to establish its utility for operator training, procedural quality assessment, and clinical outcomes.

## Data Availability

The raw data supporting the conclusions of this article will be made available by the authors, without undue reservation.
