# Supplementary material for: Mechanistic insights into the learning curve of cryoballoon ablation: ablation temperature mediates the effect of operator experience on radiation exposure
Source: Front Cardiovasc Med. 2026 Aug 27;13:1931483. doi: 10.3389/fcvm.2026.1931483 (PMC13519878; doi:10.3389/fcvm.2026.1931483)
Supplement: Supplementary file 1 [file Datasheet1.docx]

**SUPPLEMENTARY MATERIAL**

*Mechanistic Insights Into the Learning Curve of Cryoballoon Ablation: Ablation Temperature Mediates the Effect of Operator Experience on Radiation Exposure*

# **Supplementary Methods**

## **Variable Selection: LASSO Regression**

Variable selection was performed using LASSO regression implemented in the *glmnet* package (R) applied to 20 multiply-imputed datasets generated by the *mice* package (predictive mean matching for continuous variables; logistic regression for binary variables). Ten-fold cross-validation was performed for each imputed dataset, and the lambda.1se criterion was used to select the most parsimonious model within one standard error of the minimum cross-validation error. Variables selected in ≥50% of imputed datasets were retained for the multivariable model. Supplementary Figure S2 displays the cross-validation error curve and coefficient path for the primary imputed dataset.

## **Mediation Analysis: Technical Details**

The causal mediation analysis was implemented using the *mediation* package (version 4.5.0) in R.^24^ The mediator model regressed worst PV minimum temperature on learning curve phase and nine covariates (age, sex, BMI, AF type, stroke, LAD, EF, heparin dose, and hyperlipidemia). The outcome model regressed log-transformed radiation dose on learning curve phase, worst PV minimum temperature, and the same nine covariates. Bootstrap confidence intervals were based on 1,000 resamples using the quasi-Bayesian approximation. Sensitivity analysis for unmeasured confounding was evaluated using the rho parameter; ACME remained statistically significant and directionally consistent at rho values up to 0.15. The assumption of sequential ignorability (no unmeasured confounders of the mediator-outcome relationship conditional on the treatment and observed covariates) is required by this method and was not directly testable in the observational data.

# **Supplementary Tables**

**Supplementary Table S1.** Multivariable linear regression for log-transformed procedural time. Adjusted R² = 0.562. SE, standard error; BMI, body mass index; PV, pulmonary vein; AF, atrial fibrillation. Significance: *p < 0.05; **p < 0.01; ***p < 0.001.

| **Variable** | **β** | **SE** | **exp(β)** | **P value** | **Sig.** |
| --- | --- | --- | --- | --- | --- |
| Age (per year) | −0.001 | 0.001 | 0.999 | 0.105 |  |
| Male sex | 0.006 | 0.013 | 1.006 | 0.667 |  |
| Persistent AF type | 0.052 | 0.015 | 1.053 | <0.001 | *** |
| Left atrial diameter (per mm) | 0.003 | 0.001 | 1.003 | 0.014 | * |
| BMI (per kg/m²) | 0.001 | 0.002 | 1.001 | 0.767 |  |
| Hyperlipidemia | −0.039 | 0.018 | 0.962 | 0.036 | * |
| Stroke history | 0.060 | 0.023 | 1.062 | 0.008 | ** |
| Ejection fraction (per %) | 0.000 | 0.002 | 1.000 | 0.989 |  |
| Heparin dose (per U) | 0.000006 | 0.0000018 | 1.000006 | <0.001 | *** |
| Worst PV min temp (per °C) | 0.006 | 0.001 | 1.006 | <0.001 | *** |
| Plateau phase (vs. early) | −0.221 | 0.013 | 0.779 | <0.001 | *** |

**Supplementary Table S2.** Multivariable linear regression for log-transformed fluoroscopy time. Adjusted R² = 0.424.

| **Variable** | **β** | **SE** | **exp(β)** | **P value** | **Sig.** |
| --- | --- | --- | --- | --- | --- |
| Age (per year) | −0.001 | 0.001 | 0.999 | 0.164 |  |
| Male sex | −0.028 | 0.019 | 0.972 | 0.154 |  |
| Persistent AF type | 0.020 | 0.021 | 1.020 | 0.361 |  |
| Left atrial diameter (per mm) | 0.007 | 0.002 | 1.007 | <0.001 | *** |
| BMI (per kg/m²) | 0.003 | 0.003 | 1.003 | 0.357 |  |
| Hyperlipidemia | −0.096 | 0.026 | 0.908 | <0.001 | *** |
| Stroke history | 0.120 | 0.033 | 1.127 | <0.001 | *** |
| Ejection fraction (per %) | −0.003 | 0.002 | 0.997 | 0.220 |  |
| Heparin dose (per U) | 0.000006 | 0.0000025 | 1.000006 | 0.014 | * |
| Worst PV min temp (per °C) | 0.010 | 0.001 | 1.010 | <0.001 | *** |
| Plateau phase (vs. early) | −0.268 | 0.018 | 0.765 | <0.001 | *** |

# **Supplementary Figures**

**Supplementary Figure S1.** Model diagnostic plots for the primary multivariable linear regression (outcome: log-transformed radiation dose). (A) Residuals vs. fitted values. (B) Normal Q-Q plot. (C) Scale-location plot. (D) Cook’s distance vs. leverage. No major violations of linearity, normality, or homoscedasticity were identified.

**Supplementary Figure S2.** LASSO regression variable selection. (A) Mean-squared error vs. log(λ) from 10-fold cross-validation. Vertical dashed lines: lambda.min and lambda.1se. (B) LASSO coefficient paths vs. log(λ); selected variables at lambda.1se are labeled.

**Supplementary Figure S3.** Subgroup analysis forest plot: effect of worst PV minimum temperature on log-transformed radiation dose across 12 predefined patient strata. Effect estimates are exp(β) with 95% confidence intervals. All interaction p-values were non-significant (p > 0.05 for all).
